# The mediating and moderating role of social support on the relationship between psychological well-being and burdensomeness among elderly with chronic illness: community nursing perspective

**DOI:** 10.1186/s12912-025-02743-4

**Published:** 2025-02-10

**Authors:** Shaimaa Mohamed Amin, Mahmoud Abdelwahab Khedr, Ahmed Farghaly Tawfik, Mohamed Gamal Noaman Malek, Ayman Mohamed El-Ashry

**Affiliations:** 1 Lecturer of Community Health Nursing, Faculty of Nursing Damanhour, Damanhour University, Damanhour, Egypt; 2 Psychiatric and Mental Health Nursing Department, Faculty of Nursing, Alexandria University, Alexandria, Egypt; 3 Lecturer of Nursing Administration, Faculty of Nursing, Beni-suef University, Beni-suef, Egypt; 4 Lecturer of Community Health Nursing, Faculty of Nursing, Minia University, Minia, Egypt

**Keywords:** Psychological well-being, Social support, Burdensomeness, Elderly, Chronic illness, Mediating role, Moderating role, Path analysis

## Abstract

The increasing prevalence of chronic illnesses among the elderly affects their physical and psychological well-being, contributing to emotional burdens and feelings of burdensomeness. This study aims to investigate the mediating and moderating role of social support in the relationship between psychological well-being and burdensomeness among elderly individuals with chronic illnesses. A cross-sectional descriptive design was employed, involving 311 participants aged 60 and older, recruited through purposive sampling. Data were collected using validated instruments via structured interviews conducted from June to August 2024. The results indicate that perceived burdensomeness has a strong negative effect on psychological well-being (*r* = -0.654). Social support significantly mediates this relationship, with higher social support associated with lower burdensomeness (β = -0.646) and improved psychological well-being (β = 0.318). Strengthening social support networks can mitigate feelings of burdensomeness and promote mental health, ultimately enhancing the quality of life for older adults facing chronic health challenges.

## Introduction

The growing prevalence of chronic illnesses among the elderly presents significant challenges, impacting both physical health and psychological well-being. Chronic illnesses such as diabetes, cardiovascular diseases, and respiratory conditions require long-term management and often lead to substantial emotional and psychological burdens. These burdens are exacerbated by the frequent limitations imposed on daily activities and the necessity for ongoing medical treatments and lifestyle adjustments [1, 2]. In this context, understanding the role of social support in mitigating these psychological burdens and enhancing overall well-being is crucial.

While existing literature underscores the positive impact of social support on psychological well-being, previous studies may not have comprehensively examined the mediating and moderating mechanisms that underlie this relationship, particularly among elderly populations with chronic illnesses [3, 4]. For example, pathways such as demographic data, perceived stress, coping styles, or health behaviors as mediators still need to be explored. However, a study by Vaux (1885) examined the relationship between demographic factors and social support among older adults. his research revealed that social support networks are influenced by various demographic variables, including age, gender, and marital status. he found that older adults tend to have smaller but more emotionally close social networks than younger individuals [5].

Additionally, women were observed to maintain more extensive and more diverse social networks than men, often providing and receiving more social support. Marital status also played a significant role, with married individuals reporting higher levels of support, primarily from their spouses, whereas unmarried individuals relied more on friends and extended family. These findings underscore the importance of considering demographic nuances when assessing social support systems in the elderly population [5].

## The role of Social Support

Social support, defined as the perception or reality of assistance received from one’s social network, is widely recognized as a critical factor in promoting psychological health. It encompasses various forms of assistance, including emotional support (expressions of empathy and caring), informational support (providing advice and information), instrumental support (tangible aid and services), appraisal support (constructive feedback and affirmation), and companionship support (spending time together in leisure activities) [6, 7]. These forms of support collectively contribute to an individual’s sense of belonging and self-worth. Numerous studies have established the positive effects of social support on mental health, particularly in reducing symptoms of depression and anxiety, enhancing life satisfaction, and promoting overall psychological resilience [6, 7]. Additionally, related constructs such as emotional intelligence—the ability to recognize and manage emotions—and resilience—the capacity to recover from difficulties—further highlight the importance of social support in maintaining psychological well-being [7]. High levels of social support can reduce perceived stress, which in turn leads to better mental health outcomes, including reduced anxiety and depression [8, 9].

Social support, encompassing emotional, informational, instrumental, appraisal, and companionship forms, remains central to psychological resilience and stress mitigation [6, 7]. However, traditional models often overlook mediating pathways, such as Social support, which may alleviate perceived stress and indirectly enhance mental health outcomes like reduced depression and anxiety [8, 9]. Emotional intelligence moderates the impact of chronic illness, influencing how individuals perceive and utilize social support. Resilience, in turn, mediates the relationship between stressors and mental health outcomes [7]. Positive social support encourages adherence to medical regimens and health-promoting behaviors, indirectly affecting physical and psychological health [6].

### Psychological Well-Being and Burdensomeness in the Elderly

Psychological well-being in the elderly is a multi-faceted construct that includes emotional regulation, life satisfaction, and the absence of significant mental health issues. Chronic illnesses often threaten this well-being by introducing persistent stressors related to health deterioration, reduced functional capacity, and increased dependency on others. These stressors can lead to feelings of burdensomeness, where individuals perceive themselves as a burden to their family and friends. Such perceptions are linked to increased depressive symptoms, lower self-esteem, and even suicidal ideation [8, 10].

Social support plays a pivotal role in alleviating the psychological burdens associated with chronic illnesses. It helps individuals cope with stress by providing emotional comfort, practical assistance, and a sense of stability. The presence of a supportive network can significantly reduce the feelings of isolation and loneliness that often accompany chronic illnesses, thereby enhancing overall well-being [9, 11, 12].

### Cultural and contextual considerations

The sources and effectiveness of social support can vary significantly across different cultural contexts. For example, in Chinese culture, family support is often the primary source of social support for the elderly, driven by strong filial piety norms. In contrast, in Western cultures, friend support and community networks might play a more substantial role. Understanding these cultural nuances is essential for designing effective support interventions tailored to specific populations [13, 14].

The significance of social support in Arab culture is deeply rooted in its collectivist nature, which emphasizes family cohesion and community interconnectedness. Research indicates that elderly individuals in Arab societies often depend on extended family networks for both emotional and practical support, a practice that is culturally endorsed and expected. Religious and cultural norms further reinforce the importance of caring for the elderly, viewing it as a moral and spiritual duty [15, 16].

However, cultural stigmas surrounding the pursuit of external help can create challenges. Feelings of shame or dishonor may prevent the elderly from fully utilizing available support systems. For example, studies conducted in various Arab countries highlight the reliance on family as the primary source of elder care, while also noting socio-demographic changes that may hinder this traditional support system. By integrating examples from specific regions, such as the study by Hussein and Ismail, we can gain a more comprehensive understanding of these cultural dynamics [17, 18].Hussein and Ismail’s study underscores the urgent need for comprehensive policies to support the ageing population in Arab countries. It highlights the critical role of family caregivers, particularly women, and the challenges they face due to socio-demographic changes, emphasizing the importance of community-based support systems and social policies to enhance elderly care [18].

### Theoretical frameworks

Several theoretical models explain how social support influences psychological well-being. The stress-buffering hypothesis and self-determination theory (SDT) theoretical frameworks are acknowledged, but their direct application to the study’s focus on burdensomeness and social support requires further justification. These models were selected because they uniquely address the nuanced dynamics between perceived burdensomeness and social support in psychological well-being.

The stress-buffering hypothesis [19, 20] highlights how social support mitigates the adverse effects of stress by providing emotional, informational, instrumental, and appraisal support. This is particularly relevant to burdensomeness, as emotional support may alleviate feelings of being a burden, while appraisal support can help individuals reframe such perceptions in a more positive light. Informational and instrumental support further contribute by offering tangible assistance and practical guidance, reducing the stressors that may exacerbate burdensomeness.

SDT [21, 22] focuses on fulfilling the basic psychological needs of autonomy, competence, and relatedness. These needs are particularly critical in the context of burdensomeness. For example, social support that fosters autonomy can empower individuals to feel less dependent and burdensome. Support that enhances competence can counter feelings of inadequacy while fostering relatedness directly addresses the need for connection, which is often strained when individuals perceive themselves as burdensome.

These frameworks were chosen over others because they comprehensively capture the mechanisms by which social support can influence burdensomeness and psychological well-being. The stress-buffering hypothesis provides a lens to understand how support mitigates stress-related outcomes, whereas SDT offers insights into how support satisfies fundamental psychological needs. Together, they provide a multidimensional perspective, emphasizing both the reduction of stress and the promotion of intrinsic well-being [19–22].

### Significance of the study

This study aims to elucidate the complex relationships between social support, psychological well-being, and burdensomeness among the elderly with chronic illnesses. By examining both mediating and moderating pathways, it seeks to provide a comprehensive understanding of how social support can mitigate the negative psychological impacts of chronic illness. Given the aging global population and the increasing prevalence of chronic illnesses, such insights are vital for public health strategies and policies aimed at supporting this vulnerable demographic. This study aims to investigate the mediating and moderating role of social support on the relationship between psychological well-being and burdensomeness among elderly with chronic illness.

### Subjects & methods

#### Study design and setting

A cross-sectional descriptive research design was employed for this study, adhering to the Strengthening the Reporting of Observational Studies in Epidemiology (STROBE) Checklist. The research was conducted at the geriatric outpatient clinics of Minia University Hospital, which are instrumental in managing chronic diseases among older adults. These clinics offer comprehensive assessments, including evaluations of cognitive function and daily living activities, and focus on common geriatric conditions such as hypertension, diabetes, and heart disease. Personalized care plans and regular monitoring aim to enhance patients’ quality of life.

### Sample size and study population

Participants eligible for this study were adults aged 60 years and older who have been diagnosed with at least one chronic illness, such as diabetes, hypertension, or arthritis. They were required to be able to communicate effectively, willing to provide informed consent, and could be of any gender. Exclusion criteria encompassed individuals below the age of 60 years, those without a diagnosed chronic illness, and individuals experiencing severe cognitive impairment that could impair their ability to participate meaningfully or comprehend study procedures. Additionally, participants unable to communicate proficiently even with assistance, were excluded. Refusal or inability to provide informed consent also led to exclusion from the study to maintain ethical standards and ensure voluntary participation.

A power analysis for a two-tailed one-sample t-test indicated that the minimum sample size to yield a statistical power of at least 0.95 with an alpha of 0.05 and a medium effect size (d = 0.2) is 327 [23].However, The final sample included 311 older adults. While our original calculation required a sample of 327 participants, the final sample of 311 participants still meets the threshold for adequate statistical power. Using G*Power software, we recalculated the power for our mediation and moderation analysis, accounting for the reduced sample size. The updated power analysis indicated that, with a sample of 311 participants, the study maintains sufficient power (above 0.80) to detect medium effect sizes (f² = 0.15) at a 5% significance level.

### Sampling technique and recruitment

**Fig. 1:**
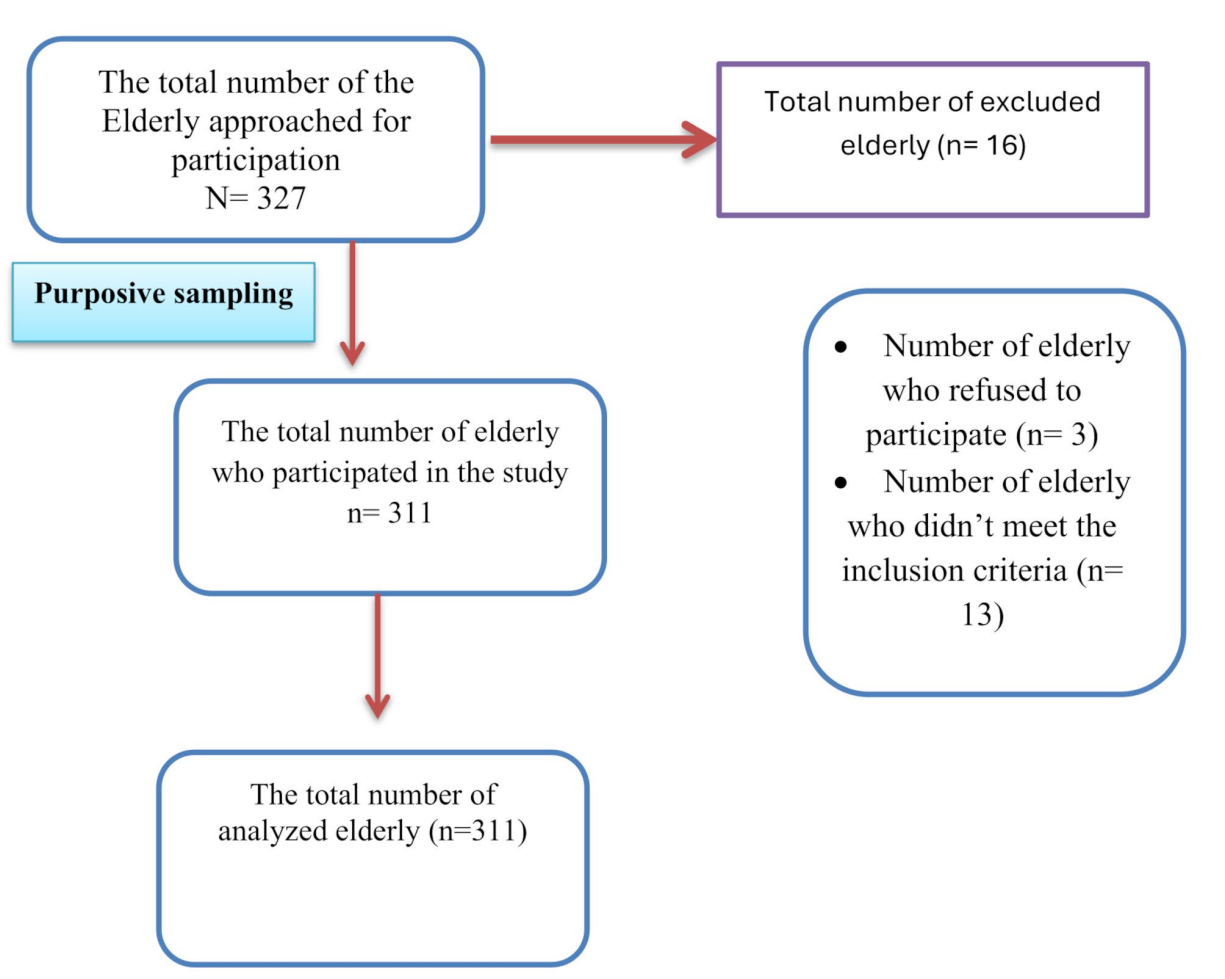
Participant’s recruitment flowchart

Purposive sampling was employed in this study to align with its research objectives, which necessitated the recruitment of older adults possessing specific characteristics pertinent to the study variables. This approach ensured the inclusion of participants who met the predefined criteria essential for examining the relationship between psychological well-being, burdensomeness, and social support. Initially, 327 older adults were invited to participate. Of these, three declined, and 13 were excluded for not meeting the inclusion criteria. The final sample included 311 older adults, whose responses were analyzed for the study (Fig. 1).

### Measurement of interests

The study involved multiple variables and their interactions, each contributing to the understanding of the factors influencing psychological well-being in elderly individuals with chronic diseases. Burdensomeness, as the independent variable, was measured using the Perceived Burdensomeness Scale (PBS), which evaluates the degree to which individuals perceive themselves as a burden to others. Comorbidities were assessed through a checklist of chronic conditions reported by the participants and were included as a control variable due to their potential influence on both psychological well-being and social support. Social support, serving as a mediator variable, was assessed using the Multidimensional Scale of Perceived Social Support (MSPSS), which measures perceived support from family, friends, and significant others. This variable was included to explore its mediating role in the relationship between burdensomeness and psychological well-being. In addition, the subdimensions of social support, family support, friends support, and significant other support were analyzed separately within the broader social support framework. Psychological well-being, the dependent variable, was measured using Ryff’s Psychological Well-Being Scale (PWBS), which assesses various aspects of well-being, including autonomy, personal growth, self-acceptance, life purpose, mastery, and environmental mastery. Finally, family type, categorized as nuclear or extended, was included as a control variable to account for any potential influence of family structure on psychological well-being.

### Older adults’ demographic and health form

This form covers a wide range of aspects, including socio-demographic information such as age, gender, education level, marital status, residence, family structure, employment status, and income. It also explores medical history, including types of chronic diseases and duration of diagnosis. Additionally, lifestyle factors such as exercise habits and risk taking behaviors like smoking are assessed.

### The geriatric feelings of Burdensomeness Scale (GFBS)

The Geriatric Feelings of Burdensomeness Scale (GFBS), developed by [24], measures feelings of burdensomeness specifically among elderly populations. The scale comprises 25 items rated on a five-point Likert scale, from strongly disagree 1 to strongly agree 5. Total scores range from 25 to 125, with higher scores indicating greater feelings of burdensomeness. The scale has demonstrated high internal consistency, with a Cronbach’s alpha of 0.97. Construct validity was confirmed through exploratory and confirmatory factor analyses, establishing the GFBS as a reliable and valid measure for assessing burdensomeness among the elderly. In this study, the GFBS also showed strong reliability, with a Cronbach’s alpha of 0.824. After translating the scale from English to Arabic, an exploratory factor analysis was conducted to validate the instrument. The factor loadings ranged from 0.442 to 0.829 before rotation and improved to a range of 0.580 to 0.953 after varimax rotation, all exceeding the 0.35 threshold and collectively explaining 68.606% of the total variance.

### Multidimensional scale of perceived social support (MSPSS)

This scale is a widely used psychometric instrument designed to measure perceived social support from three distinct sources: family, friends, and significant others. Developed by [12], the MSPSS has been validated and utilized in various research and clinical settings due to its strong psychometric properties. The MSPSS comprises 12 items, with four items allocated to each of the three subscales: Family Support, Friends Support, and Significant Other Support. Each item is rated on a 7-point Likert scale ranging from 1 (very strongly disagree) to 7 (very strongly agree).The total score of the MSPSS ranges from 12 to 84, with higher scores indicating greater perceived social support. Each subscale score ranges from 4 to 28, allowing for the assessment of specific support sources. The Multidimensional Scale of Perceived Social Support (MSPSS) categorizes the level of perceived social support into three distinct ranges based on the total score obtained from the 12 items. Scores ranging from 12 to 35 indicate a low level of perceived support. Scores between 36 and 60 reflect a medium level of perceived support, .Lastly; scores from 61 to 84 denote a high level of perceived support. These categories help in understanding the extent to which individuals feel supported by their social networks and can guide interventions to enhance social support where needed. The scale demonstrates high internal consistency, with Cronbach’s alpha values typically exceeding 0.90 for the overall scale and each subscale. Construct validity has been confirmed through factor analysis, supporting the three-factor structure of the MSPSS. Additionally, the MSPSS has shown strong convergent validity, correlating well with other measures of social support, and discriminant validity, indicating that it measures distinct constructs. In the present study, the Multidimensional Scale of Perceived Social Support (MSPSS) demonstrated strong reliability, with a Cronbach’s alpha of 0.88. After translating the scale into Arabic, content validity was assessed through exploratory factor analysis, which revealed satisfactory loadings both before and after varimax rotation. These factors collectively accounted for 78.151% of the total variance, confirming the robust construct validity of the scale. Additionally, the Kaiser-Meyer-Olkin measure and Bartlett’s test of sphericity supported the suitability of the data for factor analysis, further endorsing the scale’s validity for Arabic-speaking populations.

### The 18-item Swedish version of Ryff’s psychological wellbeing scale

The 18-item version of Ryff’s Psychological Wellbeing Scale, originally developed by Ryff and Keyes in 1995, has been adapted into a streamlined Swedish version by [25]. This adaptation focuses on assessing various dimensions of psychological well-being using both Classical Test Theory (CTT) and Item Response Theory (IRT). The scale includes 18 items distributed across six subscales: Positive Relations with Others (3 items), Environmental Mastery (3 items), Self-Acceptance (3 items), Autonomy (3 items), Personal Growth (3 items), and Purpose in Life (3 items). Each item is rated on a Likert scale ranging from 1 (strongly disagree) to 6 (strongly agree). The scale comprises ten positively scored items 1, 3, 4, 5, 6, 8, 11, 12, 13, 15 and eight negatively scored items 2, 7, 9, 10, 14, 16, 17, 18. The total score ranges from 18 to 108, with higher scores indicating greater well-being. The Swedish version of the scale has demonstrated acceptable reliability, with Cronbach’s alpha coefficients ranging from 0.48 to 0.80 across the subscales and 0.79 for the total scale [26]. In the current study, the scale exhibited excellent internal consistency, with a Cronbach’s alpha coefficient of 0.88. Additionally, after translating the scale into Arabic, the researchers conducted an exploratory factor analysis to assess its validity. The factor loadings ranged from 0.442 to 0.829 before rotation and improved to a range of 0.580 to 0.953 after varimax rotation. All factor loadings exceeded the 0.35 threshold, collectively explaining 68.606% of the total variance.

### Study procedures

#### Tool preparation &pilot study

The research instruments, including the GFBS, MSPSS, and the 18-item Swedish version of Ryff’s Psychological Wellbeing Scale, were meticulously translated into Arabic by bilingual experts fluent in both English and Arabic to ensure accuracy and cultural appropriateness. To validate the translations, a back-translation into English was performed by an independent team of bilingual experts who were not involved in the initial translation. This procedure was designed to maintain neutrality and minimize bias. The team of two bilingual translators independently translated the Arabic versions back into English, ensuring linguistic equivalence and identifying any discrepancies or misinterpretations related to cultural nuances.

After completing the back-translation, the original researchers compared the back-translated versions with the original English instruments to address any inconsistencies. If discrepancies were found, the translation was refined to ensure alignment and accuracy. Face validity assessments were then conducted, with expert panels reviewing the translated tools to ensure they captured the intended constructs in the Arabic context. Feedback from potential participants confirmed the clarity, relevance, and cultural appropriateness of the translated items. Reliability was assessed using Cronbach’s alpha to ensure internal consistency.

A rigorous process of cultural adaptation was also implemented, including consultations with local experts and incorporating community feedback to ensure the scales reflected the values, norms, and family structures common in the Arabic-speaking community. For example, the perception of burdensomeness may be shaped by family dynamics and intergenerational relationships, which differ from Western conceptualizations.

The pilot study, conducted with 30 older adults, assessed the clarity, relevance, and reliability of the instruments. Feedback from participants and reviewers indicated that the instructions, item wording, and response options were clear and comprehensible, with no issues of misinterpretation. The internal consistency of the scales in the pilot sample met acceptable thresholds, confirming their reliability. The absence of required modifications was expected, given the thorough process of translation, back-translation, and expert review prior to the pilot study. The smooth implementation of procedures and participant adherence reinforced the feasibility of the design for the main study.

### Data collection

The data collection process began with a comprehensive orientation provided to all participants, outlining the study’s objectives and emphasizing the voluntary nature of their involvement. Researchers addressed any questions or concerns promptly and reinforced the confidentiality measures in place to foster trust in the research.

Data collection was carried out by a team of trained researchers from June to August 2024. The researchers underwent detailed training to ensure that interviews with older adults, particularly those with chronic illnesses, were conducted with sensitivity and empathy. The training covered ethical considerations, such as obtaining informed consent and maintaining confidentiality; effective communication skills, including empathetic listening and building rapport; and strategies for managing emotional distress that participants might experience during the interview process. Additionally, the training emphasized the importance of respecting participants’ autonomy, addressing sensitive topics related to chronic illness and burdensomeness, and ensuring that all interactions were supportive and respectful. Researchers were also trained to refer participants to appropriate resources if needed. This approach ensured a safe and comfortable environment for participants throughout the data collection process.

Prior to participation, each participant provided written informed consent. To maintain anonymity, participants were instructed not to include any personal identifiers on the questionnaires. Participation was entirely voluntary, and participants were free to withdraw from the study at any point. Data collection was conducted through interviews in the waiting areas of the clinics. Each interview, lasting 20 to 30 min, was scheduled between 10 a.m. and 2 p.m. from Saturday to Thursday. Recognizing the varying needs of elderly participants with chronic illnesses, we prioritized flexibility in the schedule. The chosen time window was initially designed to accommodate the participants’ availability, but we also ensured flexibility by offering alternative times outside the primary schedule for those with medical appointments or other conflicting obligations. This approach was designed to maximize participation while minimizing the disruption to the participants’ routines.

### Ethical considerations

Approval for the study was granted by the Research Ethics Committee of the Faculty of Nursing, El Minia University, Egypt (REC202471). In addition, permission was secured from the directors of the participating clinics after a thorough explanation of the study’s objectives. The research was conducted in full accordance with the ethical standards outlined in the Declaration of Helsinki, ensuring the protection of participants’ rights and well-being. Before participation, all individuals were provided with a clear, detailed explanation of the study’s aims, and written informed consent was obtained. Special care was taken to ensure that participants, particularly elderly individuals, understood the study procedures, with research assistants available to clarify any questions. For participants with potential cognitive impairments, additional safeguards were implemented. If needed, caregivers or family members were involved in the consent process to ensure that participants were capable of making informed decisions.

To protect participants’ privacy, all data were handled with the strictest confidentiality. Personal identifiers were removed, and data were stored in a secure, password-protected system accessible only to the research team. For additional security, physical records were kept in locked cabinets. Participants were also made fully aware of their right to withdraw from the study at any point without facing any consequences. These measures were designed to ensure ethical compliance, safeguard participant privacy, and provide protection for those in vulnerable groups, such as the elderly.

### Data analysis

Before entering the data into the computer, it was verified for accuracy. Data analysis and tabulation were conducted using IBM SPSS Statistics version 25.0. Descriptive statistics were employed to characterize both study participants and variables. Mean scores were calculated for numerical values. A statistically significant level was considered when *p* ≤ 0.001, and the significance level was set at *p* < 0.05. A path analysis model was created for mediation analysis using SPSS-AMOS version 26. Parametric tests were utilized as the data exhibited normal distribution according to the Kolmogorov-Smirnov test, Inter-Quartile Range, and an assessment of residual plots [27]. In IBM SPSS, outliers were adjusted to their closest normal value using Winsorizing method. Outliers for this study referred to outside the fence of Q1 – (1.5 × Interquartile range [IQR]) and Q3 + (1.5 × IQR). Multicollinearity among variables was assessed. Tolerance values were confirmed to be greater than 0.1, and the Variance Inflation Factor (VIF) was less than 3. Specifically, both social support and burdensomeness had a VIF not exceding 1.693, which is below the threshold of 3, indicating no significant multicollinearity. Homoscedasticity was assessed using standardized residual plots. All assumptions were satisfactorily met, validating the robustness of our findings. Pearson’s correlation was used to analyze bivariate correlations between the study variables and their sub-dimensions. Furthermore, Andrew F. Hayes’ Process Macro was utilized as a data analysis approach. This approach is based on ordinary least squares regression and the bias-corrected bootstrapping method developed by [28]. It is recognized for its superior statistical power compared to other confidence interval analyses [29].

## Results

Table 1 displays the personal characteristics of study participants. large participants fall within the **60-<65 age range** (47.0%). Gender distribution is approximately equal between males (48.9%) and females (51.1%). Approximately two-thirds of participants are **married** (64.3%), and reside in **urban areas** (64.6%). A large proportion of study subjects have completed secondary education (45.3%). Retired (57.7%), and with enough income (62.4%). The distribution between **nuclear families** (40.2%) and **extended families** (59.2%) is slightly twisted to extended families. Most participants didn’t smoke (71.4%) and didn’t have regular exercise (83.3%). The most common comorbidity among study participants is diabetes mellitus, affecting 15.4% of participants, followed by cardiovascular diseases (10.9%) and chronic respiratory diseases (12.5%). Multimorbidity, or the presence of multiple chronic conditions, is prevalent in 40.8% of participants, highlighting a significant burden of disease among this group. In terms of the duration since diagnosis with a chronic illness, the majority of participants (60.8%) have lived with their condition for more than five years, indicating that chronic conditions in this population tend to persist over time.

**Table 1:** Distribution of the study participants according to their characteristics (*n* = 311)

Variable	Category	Frequency	Percent
**Age**	60-	146	47.0
	65-	98	31.5
	70±	67	21.5
**Gender**	Male	152	48.9
	Female	159	51.1
**Social status**	Single	7	2.3
	Married	200	64.3
	Divorced	11	3.5
	Widow	93	29.9
**Education level**	Basic	77	24.8
	Secondary	141	45.3
	University	93	29.9
**Residence**	Urban	201	64.6
	Rural	110	35.4
**Job**	self-employed	36	11.6
	Retired	180	57.9
	House wife	53	17.0
	Manual workers	33	10.6
	Farmer	9	2.9
**Income**	Enough	194	62.4
	Not enough	54	17.4
	Enough & Save	63	20.3
**Cigarette Smoking**	Yes	89	28.6
	No	222	71.4
**Regular exercise**:	Yes	52	16.7
	No	259	83.3
**Type of family**	Nuclear	125	40.2
	Extended	186	59.8
**Comorbidities**	Diabetes Mellitus	48	15.4
	Chronic Kidney Disease	7	2.3
	Cancer	11	3.5
	Cardiovascular Diseases	34	10.9
	Chronic Respiratory Disease	39	12.5
	Neurological Disease	14	4.5
	Musculoskeletal Disease	23	7.4
	Chronic Liver Disease	8	2.6
	Multimorbidity	127	40.8
**Duration since diagnosis with a chronic disease**	Less than 5	122	39.2
	5±	189	60.8
	Total	311	100.0

Table 2 presents the descriptive statistics and pairwise correlations of the study variables and their dimensions. The table reveals strong correlations between the various dimensions of social support with coefficients ranging between (0.887 and 0.974). Also, dimensions related to psychological well-being show significant correlations with coefficients ranging between (0.144 and 0.823). burdensomeness show significant negative correlations with other study variables and dimensions, the strongest correlation is with psychological wellbeing (*r*=-0.654) and the weakest correlation is with positive relations with others (*r*=-0.257). In terms of means and standard deviations, total social support has a mean score of 57.7 (SD = 17.2), and psychological well-being has a mean score of 67.2 (SD = 10.7), suggesting moderate levels of these constructs among participants. Burdensomeness has a higher variability with a mean of 59.5 (SD = 19.9), which might indicate diverse experiences of feeling burdensome within the group.

**Table 2:** Descriptive statistics and pairwise correlations of the study variables and their dimensions (*n* = 311)

		1	2	3	4	5	6	7	8	9	10	11	12
1	**Total Social Support**												
2	Family Support	0.955^**^											
3	Friends Support	0.971^**^	0.887^**^										
4	Significant Other Support	0.974^**^	0.894^**^	0.925^**^									
5	**Psychological Wellbeing**	0.595^**^	0.577^**^	0.570^**^	0.579^**^								
6	Positive Relations Others	0.238^**^	0.258^**^	0.219^**^	0.218^**^	0.452^**^							
7	Environmental Mastery	0.572^**^	0.546^**^	0.549^**^	0.564^**^	0.727^**^	0.552^**^						
8	Self Acceptance	0.235^**^	0.238^**^	0.213^**^	0.233^**^	0.603^**^	0.011	0.144^*^					
9	Autonomy	0.513^**^	0.505^**^	0.498^**^	0.486^**^	0.823^**^	0.379^**^	0.587^**^	0.427^**^				
10	Personal Growth	0.489^**^	0.474^**^	0.459^**^	0.486^**^	0.783^**^	0.365^**^	0.575^**^	0.300^**^	0.639^**^			
11	Purpose in Life	0.388^**^	0.366^**^	0.382^**^	0.375^**^	0.766^**^	0.045	0.259^**^	0.598^**^	0.493^**^	0.466^**^		
12	**Burdensomeness**	− 0.640^**^	− 0.609^**^	− 0.627^**^	− 0.619^**^	− 0.654^**^	− 0.257^**^	− 0.487^**^	− 0.369^**^	− 0.516^**^	− 0.492^**^	− 0.539^**^	
	Mean ± SD	57.7 ± 17.2	19.8 ± 5.4	19.0 ± 6.1	18.8 ± 6.3	67.2 ± 10.7	11.6 ± 1.2	13.1 ± 3.2	10.3 ± 2.0	11.7 ± 2.2	11.5 ± 2.2	8.9 ± 4.0	59.5 ± 19.9

*. Correlation is significant at the 0.05 level (2-tailed). **. Correlation is significant at the 0.01 level (2-tailed)

**Table 3:** Moderation test results PROCESS MACRO by Andrew F. HAYES, Social support as a moderator in the relation between Burdensomeness and elderly people with chronic diseases’ psychological wellbeing

	β	se	t	*p*	LLCI	ULCI
Constant	82.97	7.8384	10.5854	0.0000	67.5478	98.3975
Burdensomeness	-0.41	0.0973	-4.1919	0.0000	− 0.5993	− 0.216
Social support	6.109	2.933	2.083	0.0381	0.3378	11.88
Int_1	0.003	0.0015	2.1704	0.0308	0.0003	0.0061
Gender	2.78	0.8140	3.4170	0.0007	1.1796	4.3833
Education	2.057	0.5790	3.5529	0.0004	0.9177	3.1964
Income	1.04	0.4989	2.0819	0.0382	0.0569	2.0206
Sport	-2/37	1.1014	-2.1521	0.0322	-4.5377	− 0.2030
Family type	-3.98	0.8749	-4.5539	0.0000	-5.7059	-2.2625
R^2^	0.598					
F	56.13					
DF1	8.00					
DF2	302.0					
P	0.000					
**Int_1**						
R^2^ change	0.0063					
F	4.71					
P	0.031					

Table 3 shows that the interaction between burdensomeness and elderly people with chronic diseases’ psychological well-being moderated by social support is significant (Int_1, β = 0.003, *p* < 0.05) when controlling for gender, education, income, sport, and family type. The results of this moderation test indicated that social support moderates the effect of burdensomeness on psychological well-being.

**Fig. 2:**
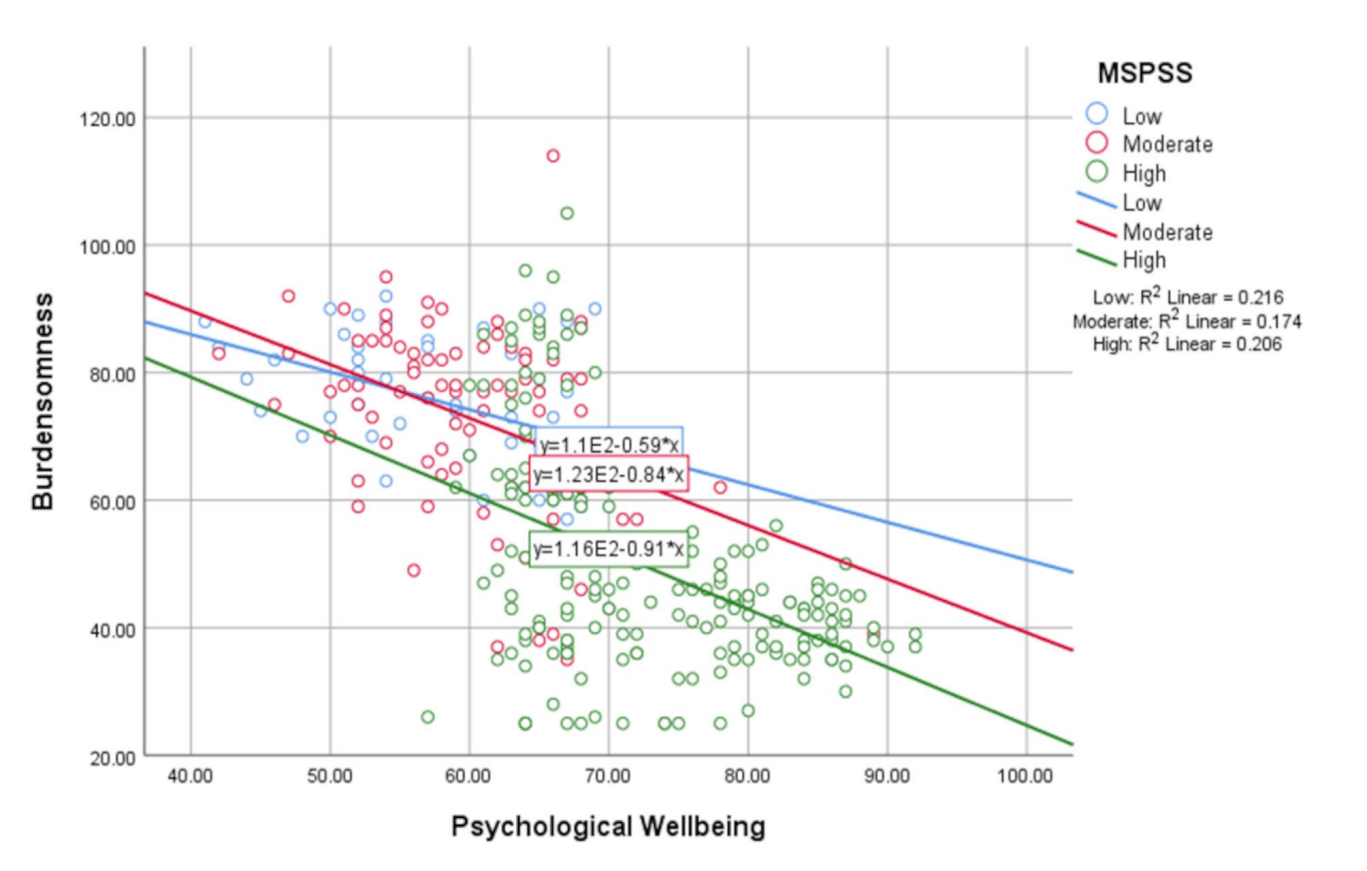
Moderation effect of Social support as a moderator in the relation between Burdensomeness and elderly people with chronic diseases’ Psychological well-being

Figure 2 presents a scatter correlation between burdensomeness and elderly people with chronic diseases’ psychological well-being categorized by social support. According to the figure as social support increases the effect of burdensomeness and elderly people with chronic diseases’ psychological well-being decreases. Elderly people with high perceived social support are less affected by burdensomeness.

**Fig. 3:**
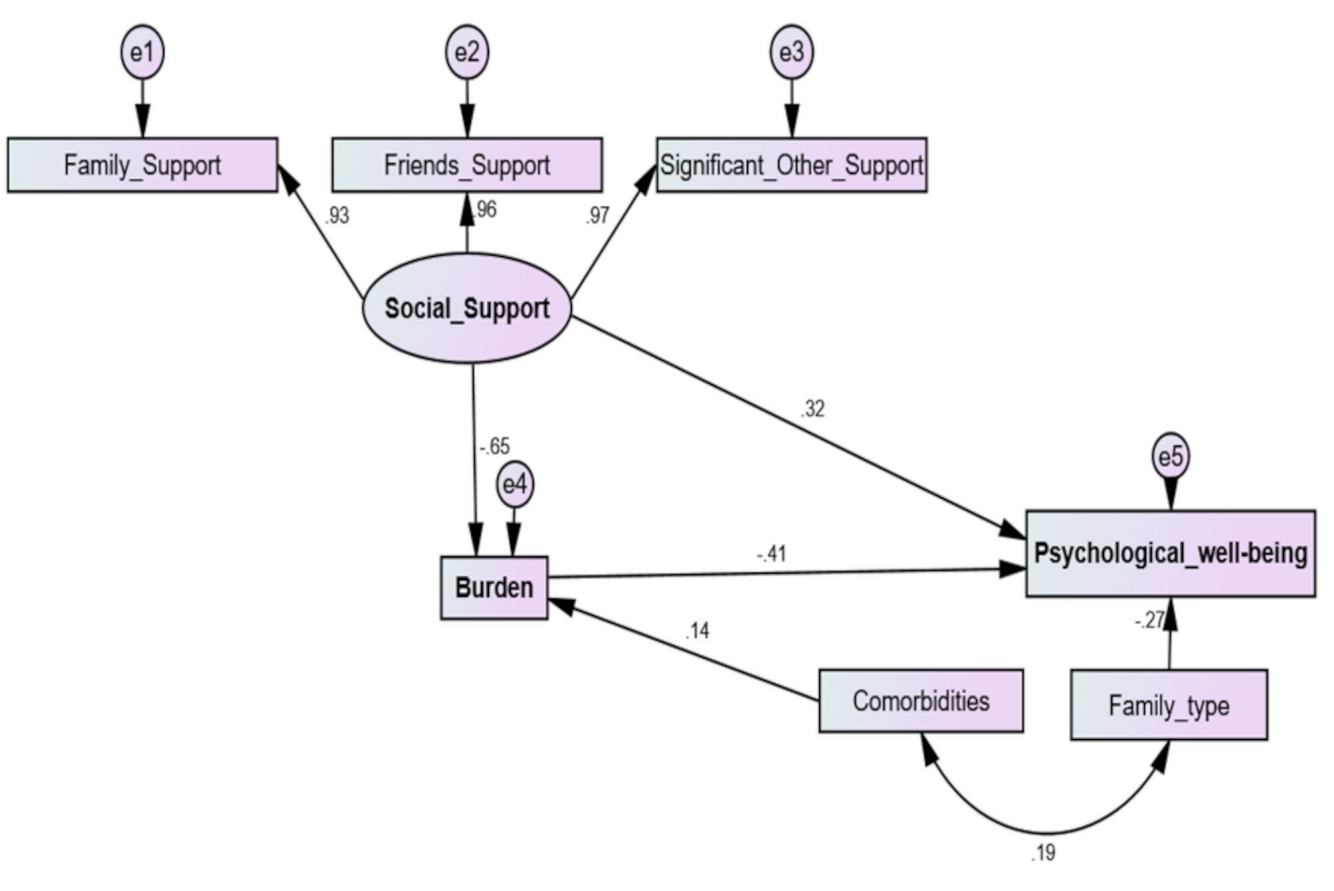
Structural equation modeling path analysis of the effect of Burdensomeness on elderly people with chronic diseases’ Psychological Well-being mediated by social support. (*n* = 311)

Model fit parameters X^2^/DF, GFI; CFI; RMSEA (1.80,0.981, 0.994, 0.051).

GFI = Goodness of Fit Index, CFI = Comparative fit index, and RMSEA = Root Mean Square Error of Approximation.

Model Chi square = 21.55, DF = 12, *p* < 0.043.

**Table 4:** Path analysis of direct and indirect effects of burdensomeness on elderly people with chronic diseases’ Psychological Well-being mediated by social support

Variable 1	Direction	Variable2	β	S.E.	C.*R*.	*P*	Significance
Burdensomeness	<---	Comorbidities	0.145	0.279	3.361	***	**Significant**
Burdensomeness	<---	Social Support	− 0.646	0.182	-14.073	***	**Significant**
Family Support	<---	Social Support	0.926				
Friends Support	<---	Social Support	0.959	0.034	33.739	***	**Significant**
Significant Other Support	<---	Social Support	0.965	0.035	34.547	***	**Significant**
Psychological Wellbeing	<---	Burdensomeness	− 0.408	0.027	-7.747	***	**Significant**
Psychological Wellbeing	<---	Family type	− 0.268	0.833	-6.749	***	**Significant**
Psychological Wellbeing	<---	Social Support	0.318	0.111	5.889	***	**Significant**

Table 4; Fig. 3. present the results from the path analysis, illustrating standardized regression weights, standard errors (SE), critical ratios (CR), and significance (p-values) of both the direct and indirect effects of burdensomeness on elderly people with chronic diseases’ psychological well-being. The statistical analysis was conducted using SPSS-AMOS. The study’s variables produced good estimates, with the comparative fit index indicating a high model fit and the root mean square approximation error also indicating a good fit. The model fit parameters met satisfactory standards (X^2^/DF, GFI; CFI; RMSEA “1.80,0.981, 0.994, 0.051”). The results of the path analysis presented show a significant impact of both burdensomeness and social support on psychological well-being. The coefficients (β) for burdensomeness and social support are both statistically significant (*p* < 0.001), indicating a strong negative effect on psychological well-being. These effects are partially mediated through social support as the direct effect is statistically significant.

## Discussion

The interplay between psychological well-being, perceived burdensomeness, and social support is crucial for elderly individuals with chronic illnesses. Enhancing social support systems could significantly improve their mental health outcomes by mitigating feelings of burdensomeness and fostering a more positive psychological state. The current study aimed to investigate the mediating and moderating role of social support on the relationship between psychological well-being and burdensomeness among elderly with chronic illness.

The examination of the study demographics reveals that a high proportion of participants aged 60 to < 65 may face transitional challenges that heighten feelings of burdensomeness and affect psychological well-being. The marital status of participants, with approximately two-thirds being married, suggests access to social support, but the quality of these relationships is crucial, as supportive marriages may lead to better mental health outcomes [30]. Additionally, gender dynamics and family type influence perceptions of burdensomeness and support availability, with women often facing greater caregiving expectations and multigenerational households providing different support dynamics [31].

The correlations among various dimensions of social support are notably strong, indicating robust interrelationships. High correlation coefficients indicate that individuals who perceive substantial support from one source, such as family, are likely to experience similar levels of support from friends or significant others. This interconnectedness is particularly crucial for elderly individuals, as it underscores the importance of a comprehensive support network in promoting mental health. Research indicates that social support can significantly buffer against psychological distress, especially in vulnerable populations [32]. Moreover, positive correlations between psychological well-being and dimensions of social support highlight that increased social support is associated with better mental health outcomes. Studies have shown that strong social networks contribute positively to life satisfaction and emotional resilience among older adults facing chronic health challenges [33].

Conversely, perceived burdensomeness shows significant negative correlations with all study variables, particularly with psychological well-being. This finding indicates that higher feelings of being a burden correlate with lower psychological health. It also highlightes that individuals who perceive themselves as burdensome often experience increased feelings of depression and anxiety, thus establishing a clear link between perceived burdensomeness and adverse mental health outcomes.

Research consistently demonstrates that perceived burdensomeness is linked to increased feelings of depression and anxiety in older adults [8]. The strong negative correlation between burdensomeness and psychological well-being supports the interpersonal theory of suicide, which posits that perceived burdensomeness and thwarted belongingness are critical factors influencing suicidal ideation and mental distress [34, 35]. This also aligns with previous research that has consistently shown that higher feelings of being a burden correlate with lower psychological health, reinforcing the idea that perceived burdensomeness is detrimental to mental health in older adults [8, 36, 37].

The mean scores for total social support and psychological well-being indicate moderate levels among participants. This suggests that while many elderly individuals experience reasonable levels of social support and psychological health, there remains considerable variability in their experiences. In contrast, the mean score for burdensomeness is higher, indicating diverse experiences regarding feelings of being a burden within the group. This variability may reflect individual differences in coping mechanisms and social contexts, as recent studies highlight the need for tailored interventions to address these perceptions [11].

The findings reveal that social support significantly moderates the relationship between burdensomeness and psychological well-being, indicating that higher levels of social support can lessen the negative effects of feeling burdensome. Specifically, elderly individuals who perceive themselves as burdensome may experience less decline in their psychological well-being if they have strong social support networks. While the current findings indicate that social support significantly moderates this relationship, it is essential to consider other potential variables that could influence these dynamics. For example, cultural norms can play a substantial role in shaping perceptions of burdensomeness and the effectiveness of social support. The current study was conducted in a region characterized by collectivist cultural values emphasizing group harmony, family interconnectedness, and communal support. In such contexts, elderly individuals often experience strong familial obligations and a sense of belonging within their community. This cultural framework can significantly shape how perceptions of burdensomeness and social support are experienced [34]. In collectivist cultures, individuals may feel less burdened by their needs due to the communal approach to caregiving. Family members are often expected to provide support, which can reduce the stigma associated with requesting help.

Additionally, caregiver availability is another critical factor that may influence this relationship. The presence of supportive caregivers can mitigate feelings of burdensomeness, providing emotional and practical assistance that enhances psychological well-being. Conversely, a lack of available caregivers may exacerbate feelings of being a burden, further impacting mental health outcomes [31]. Previous research supports this, highlighting social support as a protective factor against psychological distress, particularly among older adults with chronic illnesses [11, 38]. Additionally, the analysis accounts for various demographic factors such as gender, education, income, sport participation, and family type, all of which significantly impact psychological well-being. Gender and education are positively associated with mental health, indicating that women and those with higher educational attainment report better well-being. These results align with existing literature that emphasizes socio-demographic factors’ importance in shaping older adults’ psychological outcomes [39, 40]. Income also positively influences well-being, reinforcing the idea that financial stability contributes to better mental health.

The SEM model posits that social support directly influences psychological well-being while also exerting an indirect effect through its impact on perceived burdensomeness. This demonstrate that individuals with higher levels of social support are more likely to experience better mental health outcomes. Furthermore, the current findings indicate that social support directly and indirectly influences psychological well-being by affecting perceived burdensomeness. This relationship can be understood through Self-Determination Theory (SDT), which emphasizes the importance of fulfilling the need for relatedness, as individuals who perceive strong social support experience enhanced feelings of connection and belonging, leading to improved mental health outcomes [21]. Additionally, the stress-buffering hypothesis further elucidates our findings by positing that social support serves as a protective factor against the negative effects of stressors [19]. Our results demonstrate that social support significantly moderates the relationship between perceived burdensomeness and psychological well-being. When elderly individuals perceive themselves as burdensome, strong social support can buffer against the resulting psychological distress, thereby improving their mental health outcomes. This finding aligns with existing literature that emphasizes the protective role of social support in reducing stress and enhancing resilience among vulnerable populations.

Numerous studies have confirmed the positive association between social support and psychological well-being, indicating that social support provides essential emotional, informational, and instrumental resources that help individuals cope with stress, enhance self-esteem, and foster a sense of belonging [31, 39]. Furthermore, social support positively impacts psychological well-being indirectly by reducing feelings of burdensomeness. This relationship implies that social support can help individuals manage the challenges associated with chronic illnesses, thereby improving their mental health. Research has shown that individuals with higher levels of social support are less likely to experience depression, which is often linked to increased feelings of burden [38]. Additionally, the model examines the moderating effect of family type on the relationship between comorbidities and psychological well-being. This indicates that the impact of comorbidities on psychological well-being may vary depending on the type of family support available. For instance, individuals from supportive family environments may cope better with the challenges posed by chronic illnesses compared to those from less supportive backgrounds [41].

The path analysis reveals significant relationships among burdensomeness, social support, and psychological well-being. Specifically, burdensomeness negatively impacts psychological well-being, meaning that as individuals feel more like a burden, their mental health declines. This finding is consistent with existing literature that highlights the adverse effects of perceived burdensomeness on mental health in older adults [8, 34]. The direct effect of burdensomeness underscores the necessity of addressing these feelings in interventions aimed at enhancing mental health for elderly populations. Social support plays a critical role in this framework. The analysis indicates that social support significantly mediates the relationship between burdensomeness and psychological well-being. Higher levels of social support are linked to lower perceived burdensomeness and improved psychological well-being. This supports previous research emphasizing the protective effects of social support against psychological distress [11]. By strengthening social support networks, it may be possible to alleviate the negative impacts of burdensomeness on mental health.

In our findings, social support functions both as a moderator and a mediator and understanding the intersection of these roles is crucial for a comprehensive interpretation of our results. As a moderator, social support influences the strength and direction of the relationship between perceived burdensomeness and psychological well-being. Specifically, higher levels of social support can weaken the negative impact of perceived burdensomeness on mental health, suggesting a protective effect against psychological distress. As a mediator, social support helps explain the pathway through which perceived burdensomeness affects psychological well-being. In this context, when individuals feel more supported, their perceptions of burdensomeness are reduced, leading to improved psychological outcomes.

## Conclusion

The study findings highlight the psychological challenges faced by elderly individuals with chronic diseases, particularly in relation to their sense of burdensomeness. Social support plays a crucial role in mitigating these negative effects, but its impact varies depending on both the type of social support (e.g., emotional or instrumental) and the type of chronic illness. Emotional support, in particular, appears to have a more significant impact on psychological well-being, helping individuals cope with the emotional and psychological burdens of chronic illness. However, instrumental support may also play a key role, especially for those with physical limitations due to their conditions. The study suggests that interventions aimed at enhancing specific types of social support, tailored to the needs of individuals based on their chronic illness type, could be a more effective strategy for improving mental health outcomes. By strengthening both emotional and instrumental support networks, we can significantly improve the quality of life among elderly individuals facing chronic health challenges.

### Implications

The findings of this study have significant implications for nursing education, practice, and research, particularly in the context of elderly patients with chronic illnesses.

#### Nursing education

It is essential to incorporate training on social support mechanisms and patient-centered care into nursing curricula. Nursing students should be equipped with the skills to identify and address psychosocial challenges such as feelings of burdensomeness and insufficient social support. Courses on gerontological nursing should emphasize strategies for promoting family engagement, assessing social support systems, and understanding the mental health needs of elderly patients. Practical training may include developing family education programs that focus on enhancing communication with elderly patients and understanding their unique emotional and social needs. By incorporating these concepts, future nurses will be better prepared to provide comprehensive care that addresses the physical, emotional, and social needs of elderly individuals.

#### Nursing practice

Nurses should adopt a holistic approach that considers both the physical and psychosocial aspects of elderly care. This study highlights the importance of routinely assessing social support levels and feelings of burdensomeness in elderly patients with chronic conditions. Concrete strategies for practice may include the development of family-centered care programs, where nurses provide families with tools to enhance social support systems, such as community resources and local support groups. Promoting physical activity programs tailored to elderly individuals can also enhance well-being and strengthen social networks. Collaboration with multidisciplinary teams, including social workers, community organizations, and physical therapists, can further support these efforts, ensuring that elderly patients receive comprehensive, individualized care that improves their quality of life.

#### Nursing research

This study underscores the need for further investigation into the impact of social support and feelings of burdensomeness among elderly individuals with chronic illnesses. Future research could explore innovative nursing-led interventions aimed at improving social support and reducing burdensomeness, such as digital tools (e.g., telehealth platforms or social media groups) that can foster virtual social networks and improve mental health outcomes. Additionally, longitudinal studies could evaluate the long-term effects of social support and physical activity on psychological well-being. Comparing urban and rural settings could also provide valuable insights into how geographic context influences the availability and effectiveness of social support. Future research should further explore these cultural dimensions and consider how findings may differ in individualistic societies to provide a more comprehensive perspective on these important issues. Lastly, qualitative research capturing the lived experiences of elderly patients would provide deeper insights into their challenges and inform the development of tailored interventions.

### Strengths and limitations

This study has several strengths that enhance the validity and reliability of its findings. Firstly, the use of established and validated measurement tools, such as the Geriatric Feelings of Burdensomeness Scale and the Multidimensional Scale of Perceived Social Support, ensures that the constructs of interest were accurately assessed. The rigorous translation and validation processes for these scales into Arabic further contribute to their cultural relevance and applicability within the study population. Additionally, the cross-sectional design, while limiting in some respects, allows for a snapshot of the relationships between perceived social support, feelings of burdensomeness, and psychological well-being in older adults with chronic illnesses, providing valuable insights into their current experiences.

Despite its strengths, this study also has notable limitations. The cross-sectional design of this study limits the ability to draw causal inferences about the relationships between the variables. Since data were collected at a single point in time, it is not possible to determine the directionality or temporal sequence of the observed associations. While this design allows for the identification of correlations, it does not allow for the establishment of cause-and-effect relationships. Future research utilizing longitudinal or experimental designs would be necessary to explore potential causal pathways.

Another limitation relates to the use of Western-based instruments to measure constructs like social support. While efforts were made to translate and validate these tools for the local context, there remains a possibility of cultural bias. Certain dimensions of social support may be conceptualized or experienced differently in non-Western populations, potentially affecting the accuracy of the findings. Addressing this limitation in future research could involve the development or adaptation of culturally specific tools to better capture the lived experiences of older adults in diverse contexts.

Additionally, self-reported measures may be subject to biases, as participants could provide socially desirable responses rather than honest reflections of their feelings and experiences. Furthermore, the study was conducted in a specific clinical setting, which may limit the generalizability of the findings to broader populations or different contexts. Lastly, while the sample size was adequate for statistical analysis, it may not fully capture the diverse experiences of all older adults with chronic illnesses, necessitating future research that includes longitudinal designs and a more varied demographic to enhance understanding of these dynamics.

Finally, our results are likely most applicable to elderly individuals with chronic illnesses in similar clinical settings. Extrapolating these findings to healthier elderly populations or those in different care environments may not be appropriate due to the distinct challenges faced by our sample. Moreover, the relatively homogeneous nature of our sample limits the findings’ applicability. Factors such as socio-economic status, cultural background, and individual coping mechanisms can vary widely among broader populations, potentially leading to different experiences regarding perceived burdensomeness and social support.

## Data Availability

The datasets generated and analyzed during the current study are not publicly available due to confidentiality agreements but are available upon reasonable request from the corresponding author.
